# Heteroctanuclear Au_4_Ag_4_ Cluster Complexes of 4,5-Diethynylacridin-9-One with Luminescent Mechanochromism

**DOI:** 10.3390/molecules27072127

**Published:** 2022-03-25

**Authors:** Pei Xie, Jin-Yun Wang, Ya-Zi Huang, Xue-Meng Wu, Zhong-Ning Chen

**Affiliations:** 1 College of Chemistry, Fuzhou University, Fuzhou 350108, China; xiepei@fjirsm.ac.cn; 2 State Key Laboratory of Structural Chemistry, Fujian Institute of Research on the Structure of Matter, Chinese Academy of Sciences, Fuzhou 350100, China; jy_wang@fjirsm.ac.cn (J.-Y.W.); huangyazi@fjirsm.ac.cn (Y.-Z.H.); wuxm1@shanghaitech.edu.cn (X.-M.W.); 3 Fujian Science & Technology Innovation Laboratory for Optoelectronic Information of China, Fuzhou 350108, China

**Keywords:** cluster, gold, heteronuclear, luminescence, mechanochromism, silver

## Abstract

Two heteroctanuclear Au_4_Ag_4_ cluster complexes of 4,5-diethynylacridin-9-one (H_2_L) were prepared through the self-assembly reactions of [Au(tht)_2_](CF_3_SO_3_), Ag(tht)(CF_3_SO_3_), H_2_L and PPh_3_ or PPh_2_Py (2-(diphenylphosphino)pyridine). The Au_4_Ag_4_ cluster consists of a [Au_4_L_4_]^4−^ and four [Ag(PPh_3_)]^+^ or [Ag(PPh_2_Py)]^+^ units with Au_4_L_4_ framework exhibiting a twisted paper clip structure. In CH_2_Cl_2_ solutions at ambient temperature, both compounds show ligand fluorescence at ca. 463 nm as well as phosphorescence at 650 nm for **1** and 630 nm for **2** resulting from admixture of ^3^IL (intraligand) of L ligand, ^3^LMCT (from L ligand to Au_4_Ag_4_) and ^3^MC (metal-cluster) triplet states. Crystals or crystalline powders manifest bright yellow-green phosphorescence with vibronic-structured emission bands at 530 (568sh) nm for complex **1** and 536 (576sh) nm for complex **2**. Upon mechanical grinding, yellow-green emission in the crystalline state is dramatically converted to red luminescence centered at ca. 610 nm with a drastic redshift of the emission after crystal packing is destroyed.

## 1. Introduction

Smart luminescent materials that respond to mechanical forces show promising applications in fields such as memories, displays, sensors, and probes [1,2]. The luminescent change triggered by mechanical force is usually called luminescent mechanochromism. Various types of organic [3,4,5] and metal–organic [6,7,8] compounds have been found to show dramatic luminescent changes in emission color or intensity in response to mechanical forces such as shearing, crushing, grinding, stretching, and hydrostatic pressure. The changes in emission color or intensity under mechanical force are always reversible because the original states can be restored by solvent fuming and thermal annealing. In most cases, upon exerting a mechanical force, molecular stacking patterns undergo significant variations so that the changes in intermolecular interactions result in an alternation of photophysical properties. In fewer cases, luminescent mechanochromism arises from intramolecular contraction, deformation, or conformation variation under the action of mechanical force [3,4,9,10].

As a class of mechanically sensitive compounds, a number of d^10^ metal complexes [11,12,13,14,15,16,17,18,19,20,21,22,23] have been found to show remarkable luminescent mechanochromism due to mostly the formation or disruption of d^10^-d^10^ intermetallic interactions under the stimulus of mechanical actions such as crushing, grinding, stretching. Over the years, we have been devoted to the design of d^8^-d^10^ and d^10^-d^10^ heterometallic cluster complexes as phosphorescent materials for the use in solution-processed organic light-emitting diodes (OLEDs), taking advantage of aromatic acetylides as bridging ligands [24]. Recently, we are particularly interested in using rigid bis(acetylide) ligands for the preparation of a series of highly phosphorescent d^10^ metal cluster complexes [25,26]. In this work, we report the preparation, structure, photophysical property, and remarkable luminescent mechanochromism of two heteroctanuclear Au_4_Ag_4_ cluster complexes of 4,5-diethynylacridin-9-one (H_2_L).

## 2. Results and Discussion

**Scheme 1:**
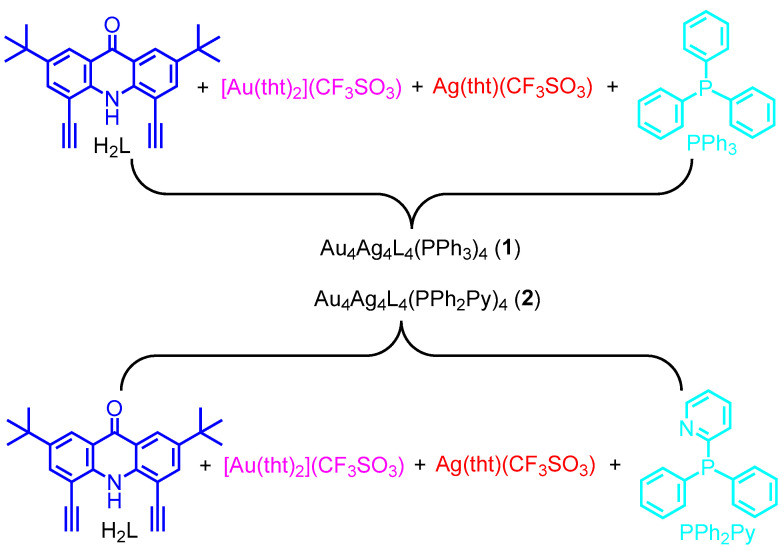
Synthetic route to Au_4_Ag_4_ cluster complexes **1** and **2**.

As shown in Scheme 1, complexes **1** and **2** were prepared by the reactions of [Au(tht)_2_](CF_3_SO_3_), Ag(tht)(CF_3_SO_3_), H_2_L, and PPh_3_ or PPh_2_Py (2-(diphenylphosphino)pyridine) in the presence of trimethylamine in 50% and 54% yields, respectively. Diffusion of Et_2_O vapor to dimethylacetamide solutions gave Au_4_Ag_4_ cluster complexes **1** and **2** as yellow crystals. They are characterized by high-resolution mass spectrometry (HRMS), ^1^H and ^31^P NMR spectroscopy, and X-ray crystallography. In the HRMS of neutral Au_4_Ag_4_ cluster complexes **1** (Supplementary Materials, Figure S1) and **2** (Figure S2), the dianionic fragment [Au_4_Ag_2_L_4_]^2−^ was, respectively, observed as a base peak for both compounds, although molecular ion peak Au_4_Ag_4_(PPh_3_)_4_L_4_ or Au_4_Ag_4_(PPh_2_Py)_4_L_4_ was not found. The observation of dianionic fragment [Au_4_Ag_2_L_4_]^2−^ with high abundance suggests that Au_4_L_4_ framework with a paper clip structure generated through gold(I)-bis(acetylide) coordination is sufficiently stable in ionization state. The ^1^H and ^31^ P NMR spectra (Figures S3–S6) indicate that Au_4_Ag_4_ cluster structures keep integrity in CD_2_Cl_2_ or DMSO-*d*_6_ solutions.

**Figure 1:**
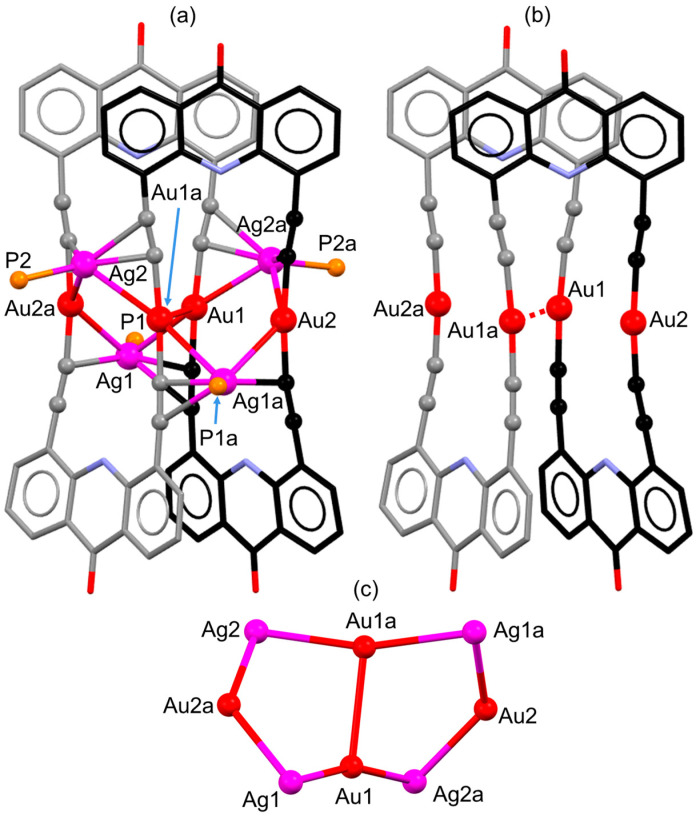
(**a**) A perspective view of Au_4_Ag_4_ complex **1** plotted from X-ray crystallography. The hydrogen atoms and *tert*-butyl groups together with the phenyl rings on phosphorous atoms were omitted for clarity. (**b**) A view showing a twisted paper clip structure of Au_4_L_4_ framework formed by gold(I)-bis(acetylide) σ-coordination. (**c**) A view of Au_4_Ag_4_ cluster showing intermetallic contacts.

As depicted in Figure 1 (complex **1**) and Figure S7 (complex **2**), the Au_4_Ag_4_ cluster complex is derived from incorporating tetranuclear [Au_4_L_4_]^4−^ with four [Ag(PPh_3_)]^+^ or [Ag(PPh_2_Py)]^+^ units through Ag−acetylide π-coordination. The Au_4_Ag_4_ cluster structure (Figure 1c) is substantially stabilized by d^10^-d^10^ intermetallic interactions because the Ag-Au (2.8455(6)–3.0488(6) Å) and Au-Au (3.1980(7)–3.2384(7) Å) distances are much shorter than the sum of van der Waals radius of Ag and Au atoms (3.35 Å) or two Au atoms (3.40 Å). As shown in Figure 1b, the Au_4_L_4_ framework formed by gold(I)-bis(acetylide) σ-coordination exhibits a twisted paper clip structure [27,28], which is further stabilized by substantial Au-Au (*d*_Au1-Au1a_ = 3.1980(7) Å for complex **1** and *d*_Au1-Au1a_ = 3.2384(7) Å for complex **2**) interaction. As depicted in Figure 1c, the Au_4_ centers are arranged in a parallelogram, whereas the Ag_4_ centers are oriented in a triangular pyramid, which is contacted through significant Ag-Au interaction. The gold(I) center is quasi-linearly bonded to two acetylide C donors with the C-Au-C angles of ca. 174°. The silver(I) center is bound to one P atom and two π-bonding acetylides with a triangle-planar geometry.

**Figure 2:**
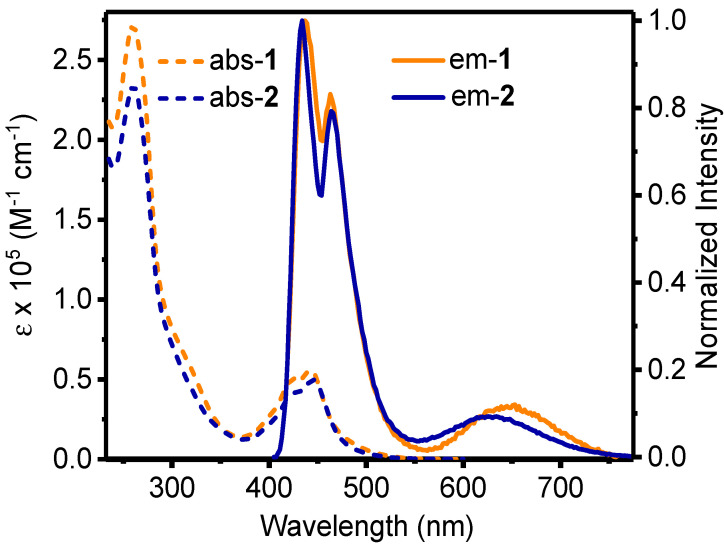
The UV−Vis absorption (dash) and normalized emission (solid) spectra of Au_4_Ag_4_ cluster complexes **1** (orange) and **2** (blue) in CH_2_Cl_2_ solutions at ambient temperature (excitation at 380 nm).

The UV-Vis absorption spectra of Au_4_Ag_4_ cluster complexes (Figure 2) display intense absorption bands peaked at 260 nm due to ligand-centered transitions. Another broadband is centered at around 440 nm, ascribed to mainly intraligand (IL) transition, ligand-to-ligand charge transfer (LLCT) transition from one L ligand to another as well as ligand-to-metal charge transfer (LMCT) transition from L ligand to Au_4_Ag_4_ core as revealed by TD-DFT studies (vide infra).

The luminescent data of complexes **1** and **2** are listed in Table 1. Upon irradiation with UV light at >220 nm, both compounds show bright luminescence in fluid solutions, solid states (crystal or powder), and doping films. As depicted in Figure 2, both compounds in CH_2_Cl_2_ solutions at ambient temperature exhibit an intense emission band peaked at ca. 463 (437sh) nm for complex **1** and 464 (434sh) nm for complex **2** together with a broadband centered at 650 nm for **1** and 630 nm for **2**. The high-energy band with the lifetime of nanosecond is due to intraligand fluorescence whereas the low-energy band with the lifetime in microsecond range is phosphorescent in character, resulting from Au_4_Ag_4_ cluster structure. The concurrent observation of intraligand fluorescence and Au_4_Ag_4_ cluster-based phosphorescence implies incomplete energy transfer from L ligands to Au_4_Ag_4_ cluster centers in fluid solutions. Relative to that of complex **1** (λ_em_ = 650 nm), the phosphorescent band of complex **2** (λ_em_ = 630 nm) shows a distinct blueshift, which is interpreted by the larger HOMO-LUMO energy gap of **2** (3.34 eV) than that of **1** (3.29 eV) upon the substitution of PPh_3_ with PPh_2_Py because introducing pyridyl results in more lowering of the HOMO than that of the LUMO level (Figure S10).

In striking contrast, only a broad phosphorescent band is, respectively, observed in the solid state and doping film without the appearance of ligand-centered emission band owing to efficient energy transfer in the aggregate state or rigid matrix. Furthermore, the phosphorescence of complexes **1** and **2** (Table 1) in crystal or crystalline state become much stronger than that in solution, ascribed to an efficient energy transfer from L ligand to metal cluster center as well as effective attenuation of non-radiative deactivation. More interestingly, the phosphorescent quantum yield of the doping film composed of 3% Au_4_Ag_4_ complex and 97% PMMA is increased to 17.3% for **1** and 15.7% for **2**, which is much higher than that in the pure solid state, owing to the overcoming intermolecular emission quenching of Au_4_Ag_4_ moieties in the aggregate state when it is diluted to a rigid matrix.

**Figure 3:**
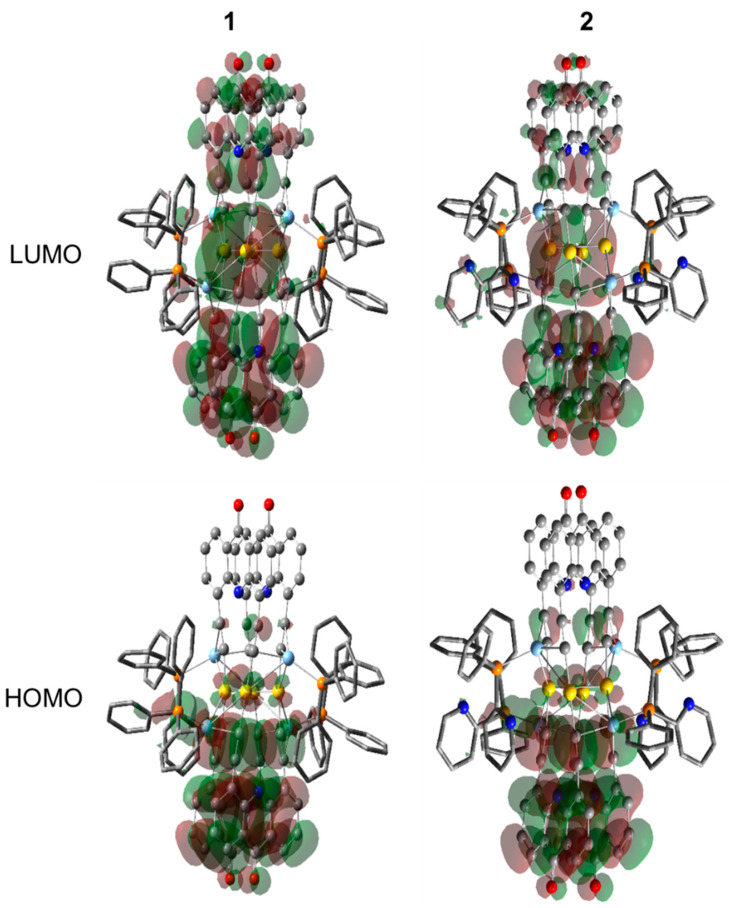
Plots of the HOMO and LUMO in triplet state (isovalue = 0.012) for Au_4_Ag_4_ Cluster Complexes **1** and **2** by TD-DFT method at the PBE1PBE-GD3 level.

To clarify the transition character and spectral origin of absorption and emission, the ground (G_0_) and the lowest-energy triplet (T_1_) state properties of complexes **1** and **2** were investigated by TD-DFT calculation. Plots of the HOMO and LUMO in G_0_ and T_1_ states are depicted in Figure S9 and Figure 3, respectively. In the G_0_ state, the HOMO (Figure S9) focuses on two of the four L ligands together with some population at Au_4_Ag_4_ core, whereas the LUMO populates mostly at another two L ligands and Au_4_Ag_4_ cluster. Thus, the lowest-energy absorption (Table S4 for **1**) originates primarily from charge transfer transition from two L to another two L ligands (LLCT) or Au_4_Ag_4_ cluster (LMCT) as well as metal cluster-centered [d→s/p] (MC) transition.

By comparison, the HOMO (Figure 3) in the lowest-energy triplet state concentrates mostly on two L ligands and the LUMO still distributes on the two L ligands as well as Au_4_Ag_4_ cluster centers with some population at another two L ligands. As a result, the lowest-energy phosphorescent emission arises from admixture of ^3^IL (L ligand) and ^3^[π(L)→s/p (Au_4_Ag_4_)] ^3^LMCT (ligand-to-metal charge transfer) transitions together with some character of ^3^[d→s/p (Au_4_Ag_4_)] ^3^MC triple state. As shown in Figure S10, a larger HOMO-LUMO energy gap for complex **2** (3.34 eV) than that of **1** (3.29 eV) rationalizes the distinct blueshift of the emission for **2** (*λ*_em_ = 630 nm) relative to that of **1** (*λ*_em_ = 650 nm) in CH_2_Cl_2_ solutions.

**Figure 4:**
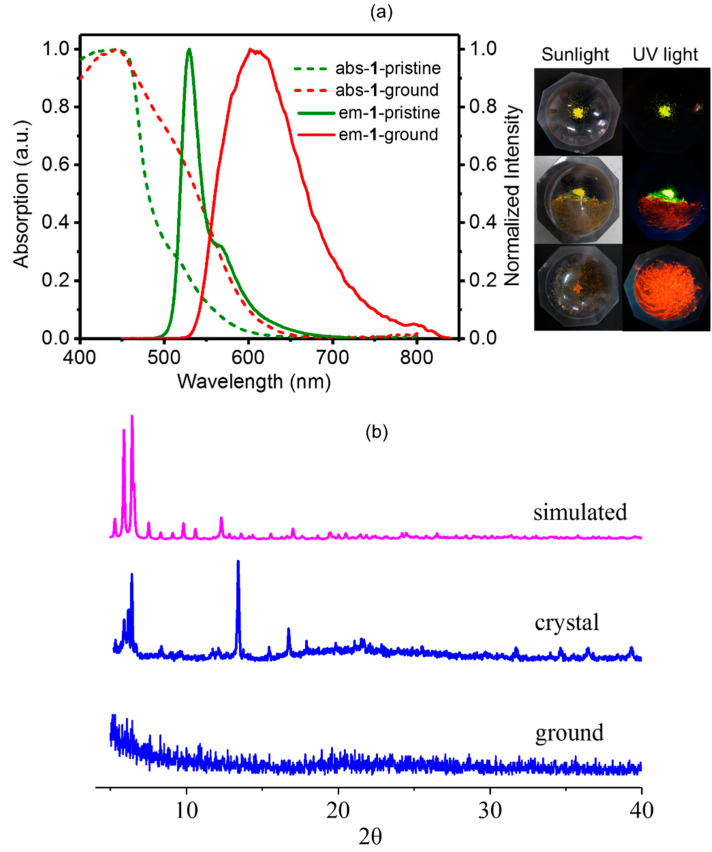
(**a**) The normalized UV-Vis absorption and emission spectra together with the images of Au_4_Ag_4_ cluster complex **1** under ambient light and UV irradiation (365 nm) before and after mechanical grinding. (**b**) The simulated and measured X-ray diffraction patterns of Au_4_Ag_4_ cluster complex **1** before and after mechanical grinding.

Intriguingly, the phosphorescent emission of Au_4_Ag_4_ complexes in the crystal state is highly sensitive to mechanical grinding. As shown in Figure 4, yellow crystals or crystalline powders exhibit bright yellow-green luminescence with an emission band peaked at 530 (568sh) nm for complex **1** and 536 (576sh) nm for complex **2**. Vibronic-structured bands are observed with vibrational progression of 1262 cm^−1^ for complex **1** and 1296 cm^−1^ for complex **2**, which is characteristic of the vibrational frequencies of the aromatic C=C modes of L ligands. This suggests that L ligands participate substantially in the emission state of Au_4_Ag_4_ complexes in solid state. Upon being mechanically ground, yellow crystals or crystalline powders become orange under ambient light with significant redshifts of low-energy absorption bands as depicted in Figure 4. Most remarkably, under irradiation UV light at 365 nm, yellow-green luminescence of crystals or crystalline powders turn strikingly into red-emitting upon mechanical grinding for both complexes **1** (Figure 4a) and **2** (Figure S13a). When mechanical grinding is exerted, the vibronic-structured emission peaks of crystals or crystalline powders at 530 (568sh) nm for complex **1** (Figure 4a) and 536 (576sh) nm for complex **2** (Figure S13a) vanish entirely, whereas a broadband centered at ca. 610 nm occurs instead with a drastic redshift of the emission relative to that before grinding. As demonstrated by X-ray diffraction (XRD) studies (Figure 4b for complex **1** and Figure S13b for complex **2**), regular crystal stacking is entirely disrupted upon mechanical grinding to induce a distinct morph conversion from crystalline to amorphous state [9,10]. On the other hand, when the ground orange samples are exposed to Et_2_O vapor, they revert perfectly to the original yellow under ambient light and red luminescence restores to yellow-green emission upon UV light irradiation. Thus, the phosphorescent mechanochromism of Au_4_Ag_4_ complexes **1** and **2** is totally reversible.

In order to ascertain the mechanochromic mechanism of Au_4_Ag_4_ complexes, complexes **1** and **2** in 3% weight percentage were uniformly dispersed to PMMA (polymethyl methacrylate) in CH_2_Cl_2_ solutions. After removal of the solvent, highly diluted solid samples in PMMA matrix are produced. Upon excitation at >300 nm, complexes **1** (Figure S11) and **2** (Figure S12) in diluted PMMA matrix display, respectively, a broad emission band centered at 606 and 592 nm with the emissive lifetime in microsecond range, which is close to the emission property in CH_2_Cl_2_ solution. Such a broad emission band is quite different from that in crystals or crystalline powder but resembles that in a mechanically ground state. Furthermore, when the diluted solid sample in PMMA matrix was mechanically pestled, only insignificant emission spectral change is observed as depicted in Figure S11 (complex **1**) and Figure S12 (complex **2**). It appears that the yellow-green phosphorescence in the crystalline state with vibronic-structured bands at 530 (568sh) nm for complex **1** and 536 (576sh) nm for complex **2** arises most likely from the rigidity effect of crystal packing or molecular stacking. When crystal packing is totally destroyed by mechanical grinding, vibronic-structured yellow-green emission is thus turned into a red-shifted and broadband similar to that observed in the solution. Some extent blueshift of the emission band in the ground powder or PMMA matrix compared with that in fluid CH_2_Cl_2_ solution is ascribable to the distinct solvation of Au_4_Ag_4_ cluster molecules.

## 3. Experimental Section

All reactions were carried out under a dry argon atmosphere using Schlenk techniques and vacuum-line systems. The solvents were dried, distilled, and degassed prior to use. Spectroscopic grade reagents for spectroscopic measurements were purchased from commercial sources. Other reagents were purchased from commercial sources without further purification. 2,7-Di-*tert*-butyl-4,5-diethynyl-9,10-dihydroacridin -9-one (H_2_L) was similarly prepared by previously reported procedure [29]. Perchlorate salts are potentially explosive and should be handled carefully.

### 3.1. Au_4_Ag_4_L_4_(PPh_3_)_4_ (***1***)

Fresh prepared [Au(tht)_2_](CF_3_SO_3_) (0.06 mmol) was obtained by mixing equivalent Ag(tht)(CF_3_SO_3_) (0.06 mmol, 20.7 mg) and Au(tht)Cl (0.06 mmol, 19.2 mg) in 10 mL CH_2_Cl_2_ with stirring for 10 min. The turbid solution was treated by centrifuge to give a clear solution, which was used directly in the next step. To the filtrate was added Ag(tht)(CF_3_SO_3_) (0.04 mmol, 13.8 mg), PPh_3_ (0.08 mmol, 21.0 mg) and H_2_L (0.08 mmol, 28.4 mg). After 5 min stirring, 0.5 mL NEt_3_ was added to the above solution and the mixture immediately turned from yellow to dark red. The stirring was kept overnight in the dark, then the solvent was removed under vacuum to give an orange solid. The solid was re-dissolved in 4 mL of dimethylacetamide (DMAc) and the clear solution was exposed to diethyl ether for slow vapor diffusion. Yellow crystals were obtained in two weeks. Yield: 50%. HRMS m/z (%): 1208.1938 (100) [Au_4_Ag_2_L_4_]^2−^ (Calcd 1208.1943). Anal. Calcd for C_172_H_152_N_4_Ag_4_Au_4_O_4_P_4_: C, 56.10; H, 4.16; N, 1.52. Found: C, 55.98; H, 4.16; N, 1.37. ^1^H NMR (400 MHz, DMSO-*d*_6_, δ): 9.95 (s, 4H), 7.75 (s, 12H), 7.70–7.43 (m, 46H), 7.42–7.32 (m, 18H), 1.16 (s, 72H). ^31^P NMR (162 MHz, DMSO-*d*_6_): 7.4 (P-Ag, br, 4P). IR (KBr, cm^−1^): 3472 m (N–H), 1635 m (C=O), 2059w (C≡C).

### 3.2. Au_4_Ag_4_L_4_(PPh_2_Py)_4_ (***2***)

This compound was prepared by the same procedure as that of complex **1** except for the use of PPh_2_Py in place of PPh_3_. Yield: 54%. HRMS m/z (%): 1208.1947 (100) [Au_4_Ag_2_L_4_]^2−^ (Calcd 1208.1943). Anal. Calcd for C_168_H_148_N_8_Ag_4_Au_4_O_4_P_4_⋅CH_2_Cl_2_: C, 53.82; H, 4.01; N, 2.97. Found: C, 53.70; H, 4.18; N, 3.19. ^1^H NMR (400 MHz, CD_2_Cl_2_, δ): 10.04 (s, 4H), 8.48 (s, 4H), 7.92 (s, 8H), 7.77 (s, 16H), 7.40 (s, 8H), 7.16 (m, *J* = 7.6 Hz, 32H), 6.91 (s, 4H), 1.01 (s, 72H). ^31^P NMR (162 MHz, CD_2_Cl_2_): 7.91 (P-Ag, br, 4P). IR (KBr, cm^−1^): 3480 m (N–H), 1640 m (C=O), 2060 w (C≡C).

### 3.3. Physical Measurements

UV-Vis absorption spectra were measured on a Perkin-Elmer Lambda 365 UV-Vis spectrophotometer. Infrared spectra (IR) were recorded on a Bruker VERTEX 70 FT-IR spectrophotometer with KBr pellets. High-resolution mass spectrometry (HRMS) was recorded on a Bruker Impact II Q-TOF mass spectrometer using dichloromethane and methanol mixtures as mobile phases. ^1^H and ^31^P NMR spectra were performed on a Bruker Avance III 400 spectrometer with SiMe_4_ and H_3_PO_4_ as internal and external references, respectively. Emission and excitation spectra and emission lifetimes in degassed solutions, solid states, and films were determined on an Edinburgh analytical instrument (FLS920 fluorescence spectrometer). Absolute quantum yields were determined by the integrating sphere (142 mm in diameter) using Edinburgh FLS920 Spectrofuorophotometer.

### 3.4. Crystal Structural Determination

Crystals suitable for X-ray crystallographic measurement were grown by vapor diffusing diethyl ether to a DMAc solution of complex **1** or **2**. The X-ray single-crystal diffraction data were collected on a Bruker D8 Venture diffractometer using IμS 3.0 microfocus source Mo-K*α* radiation (*λ* = 0.71073 Å) and PHOTON II CPAD detector. Frames were integrated with the Bruker SAINT software package (V8.38A) using a SAINT algorithm. Data were corrected for absorption effects using the multi-scan method (SADABS) [30]. The structure was solved and refined using the Bruker SHELXTL Software Package, a computer program for automatic solution of crystal structures, and refined by the full-matrix least-squares method with ShelXle Version 4.8.6, a Qt graphical user interface for the SHELXL [31]. All non-hydrogen atoms were refined anisotropically, whereas the hydrogen atoms were generated geometrically and refined using isotropic thermal parameters. CCDC 2155149-2155150 contains the supplementary crystallographic data for this paper. These data can be obtained free of charge from the Cambridge Crystallographic Data Centre via www.ccdc.cam.ac.uk/data_request/cif (accessed on 28 February 2022).

### 3.5. Computational Method

The calculations were implemented by using Gaussian 16 program package [32]. The geometrical structures as isolated molecules in the ground state and the lowest-energy triplet state were firstly optimized, respectively, by the restricted and unrestricted density functional theory (DFT) method with the gradient corrected correlation functional PBE1PBE [33,34] adding the D3 version of Grimme’s dispersion with the original D3 damping function [35] (here abbreviated as PBE1PBE-GD3). The initial structures of cluster complexes **1** and **2** were extracted from the single-crystal structural data. To save the computation time, the reduced models were used, in which the tert-butyl in L ligand were replaced by H atoms. To analyze the absorption and emission transition properties, 80 singlet and 6 triplet excited states were calculated, respectively, based on the optimized structures in the ground state and lowest-energy triplet state to determine the vertical excitation energies by time-dependent density functional theory (TD-DFT) [36,37,38] at PBE1PBE-GD3 level. In the calculation of excited states, the polarizable continuum model method (PCM) [39] with CH_2_Cl_2_ as solvent was employed. In these calculations, the Stuttgart-Dresden (SDD) basis set, which contains the f-type polarization functions (f-exponent is 1.611 for Ag and 1.050 for Au atoms) and the effective core potentials (ECPs) were used to describe the Ag and Au atoms [40,41], while other non-metal atoms of P, O, N, C, and H were described by the all-electron basis set of 6-31G**. Visualization of the frontier molecular orbitals was performed by GaussView. The contributions of fragments to the orbitals in the electronic excitation process were analyzed by the Ros and Schuit method [42] (C-squared population analysis method, SCPA) in Multiwfn 3.8 program [43].

## 4. Conclusions

Heteroctanuclear Au_4_Ag_4_ cluster complexes of 4,5-diethynylacridin-9-one were prepared in high yields by self-assembly reactions using PPh_3_ or PPh_2_Py as a co-ligand. They are composed of Au_4_L_4_ having a twisted paper clip arrangement and four [Ag(PPh_3_)]^+^ or [Ag(PPh_2_Py)]^+^ units incorporated together through Ag−acetylide π-coordination as well as substantial Au-Ag intermetallic interaction. They show both short-lived intraligand fluorescence and long-lived phosphorescence based on Au_4_Ag_4_ cluster structure in fluid solutions, but only phosphorescence is observed in crystals and doping films with much higher emissive quantum yields. When the crystals are mechanically ground, yellow-green emission with vibronic-structured bands at 530 (568sh) nm for complex **1** and 536 (576sh) nm for complex **2** becomes to red phosphorescence with a broadband centered at ca. 610 nm. The drastic redshift of the emission upon mechanical grinding is ascribed to the disruption of rigidity effect of crystal packing and alternation of molecular stacking pattern.

## Figures and Tables

**Table 1:** The UV-Vis Absorption and Luminescent Data of Complexes **1** and **2** at Ambient Temperature.

	*λ*_abs_/nm (*ε*/M^−1^ cm^−1^)	*λ*_em_ (nm)/*τ*_em_ (μs)/*Φ*_em_ (%)		
	CH_2_Cl_2_	CH_2_Cl_2_ ^a^	Solid ^b^	PMMA Film ^d^
**1**	260 (275,700), 424 (51,000), 443 (56,683)	463(437sh)/3.7ns/ ≤ 0.1	530/3.0/4.3	606/3.6/17.3
		650/1.8/1.8 ^c^	610/2.1/0.4 (G)	615/4.0/8.1 (G)
**2**	260 (233,600), 424 (41,300), 445 (50,080)	464(434sh)/4.3ns/ ≤ 0.1	536/3.0/4.8	592/4.0/15.7
		630/2.1/2.1 ^c^	610/3.5/0.4 (G)	601/5.1/7.7 (G)

^a^ Measured in degassed CH_2_Cl_2_ solutions upon excitation at 380 nm. ^b^ Measured upon excitation at 380 nm. ^c^ Measured upon excitation at 470 nm. ^d^ Consisting of 97% PMMA and 3% Au_4_Ag_4_ complex before and after mechanical grinding (G) upon excitation at 417 nm.

## Data Availability

All data needed to support the conclusions of this manuscript are included in the main text or the Supplemental Information.

## Supplementary Materials

The following supporting information can be downloaded at: https://www.mdpi.com/article/10.3390/molecules27072127/s1, Figure S1: The high-resolution mass spectrometry of complex **1**. Inset: The measured and simulated isotopic patterns; Figure S2: The high-resolution mass spectrometry of complex **2**. Inset: The measured and simulated isotopic patterns; Figure S3: The ^1^H NMR spectrum of complex **1** in DMSO-*d*_6_ solution at ambient temperature; Figure S4: The ^31^P NMR spectrum of complex **1** in DMSO-*d*_6_ solution at ambient temperature; Figure S5: The ^1^H NMR spectrum of complex **2** in CD_2_Cl_2_ solution at ambient temperature; Figure S6: The ^31^P NMR spectrum of complex **2** in CD_2_Cl_2_ solution at ambient temperature; Figure S7: (a) A perspective view of Au_4_Ag_4_ complex **2** plotted from X-ray crystallography. The hydrogen atoms and *tert*-butyl groups together with the phenyl and 2-pyridyl rings on phosphorous atoms were omitted for clarity. (b) A view showing a twisted paper clip structure of gold(I)-bis(acetylide) coordination framework; Figure S8: The plots of thermogravimetric analyses of complexes **1** and **2**; Figure S9: Plots of the HOMO and LUMO in ground state for Au_4_Ag_4_ complexes **1** and **2** by TD-DFT method at the PBE1PBE-GD3 level; Figure S10: Plots of the energy level of frontier molecular orbitals (HOMO-3~LUMO+3) based on the ground (S_0_) and lowest-energy triplet (T_1_) state structures of complexes **1** and **2**; Figure S11: The normalized emission spectra of 3% Au_4_Ag_4_ cluster complex **1** in PMMA matrix under UV irradiation (365 nm) before and after mechanical grinding; Figure S12: The normalized emission spectra of 3% Au_4_Ag_4_ cluster complex **2** in PMMA matrix under UV irradiation (365 nm) before and after mechanical grinding; Figure S13: (a) The normalized UV-Vis absorption and emission spectra together with the images of Au_4_Ag_4_ cluster complex **2** under ambient light and UV irradiation (365 nm) before and after mechanical grinding. (b) The simulated and measured X-ray diffraction patterns of Au_4_Ag_4_ cluster complex **2** before and after mechanical grinding; Table S1: Crystallographic Data for Au_4_Ag_4_ Cluster Complexes **1** and **2**; Table S2: Selective Interatomic Distances (Å) and Bonding Angles (°) of Au_4_Ag_4_ Cluster Complex **1**; Table S3: Selective Interatomic Distances (Å) and Bond Angles (°) of Au_4_Ag_4_ Cluster Complex **2**; Table S4: The Partial Molecular Orbital Compositions (%) by SCPA Approach in the Ground State and Absorption Transitions for Complex **1** in CH_2_Cl_2_ Solution, Calculated by TD-DFT Method at the PBE1PBE-GD3 Level; Table S5: The Partial Molecular Orbital Compositions (%) by SCPA Approach in the Lowest-Energy Triplet State and Emission Transitions for Complex **1** in CH_2_Cl_2_ Solution, Calculated by TD-DFT Method at the PBE1PBE-GD3 Level; Table S6: The Partial Molecular Orbital Compositions (%) by SCPA Approach in the Ground State and Absorption Transitions for Complex **2** in CH_2_Cl_2_ Solution, Calculated by TD-DFT Method at the PBE1PBE-GD3 Level; Table S7: The Partial Molecular Orbital Compositions (%) by SCPA Approach in the Lowest-Energy Triplet State and Emission Transitions for Complex **2** in CH_2_Cl_2_ Solution, Calculated by TD-DFT Method at the PBE1PBE-GD3 Level.

## Sample Availability

None of the unique compounds generated in this study are readily available.
