# Supplementary material for: Heteroctanuclear Au4Ag4 Cluster Complexes of 4,5-Diethynylacridin-9-One with Luminescent Mechanochromism
Source: Molecules. 2022 Mar 25;27(7):2127. doi: 10.3390/molecules27072127 (PMC9000280; doi:10.3390/molecules27072127)
Supplement: Supplementary file 1 [file molecules-27-02127-s001.zip › molecules-1640836-supplementary.pdf]

Supporting Information

Heteroctanuclear Au_4Ag_4 Cluster Complexes of 4,5-Diethynylacridin-9-One with Luminescent Mechanochromism

Pei Xie [1,2], Jin-Yun Wang [2], Ya-Zi Huang [2], Xue-Meng Wu [2] and Zhong-Ning Chen [1,2,3,*]

[1] *College of Chemistry, Fuzhou University, Fuzhou, Fujian 350108, China*

[2] *State Key Laboratory of Structural Chemistry, Fujian Institute of Research on the Structure of Matter, Chinese Academy of Sciences, Fuzhou, Fujian 350002, China*

[3] *Fujian Science & Technology Innovation Laboratory for Optoelectronic Information of China, Fuzhou, Fujian 350108, China*

Table S1. Crystallographic Data for Au_4Ag_4 Cluster Complexes **1** and **2**.

	**1**	**2**
empirical formula	$C_{172}H_{152}Ag_4Au_4N_4O_4P_4$	$C_{168}H_{148}Ag_4Au_4N_8O_4P_4$
formula weight	3682.19	3686.16
crystal system	monoclinic	monoclinic
space group	*C*2/*c*	*C*2/*c*
a (Å)	18.4728(11)	19.009(4)
b (Å)	39.030(2)	38.632(8)
c (Å)	23.4922(11)	23.355(5)
β (deg)	91.422(2)	93.807(9)
V (Å^3)	16932.4(16)	17113(6)
Z	4	4
F (000)	7232.0	7232.0
completeness	0.995	0.993
ρ_{calcd} (g/cm^3)	1.444	1.431
μ (mm^{-1})	3.991	3.950
radiation (λ, Å)	0.71073	0.71073
temperature (K)	150	150
GOF	1.019	1.083
R1 (F_o)^a	0.0478(10392)	0.0312(14627)
wR2 (F_o^2)^b	0.1469(15516)	0.1163(17428)

^{a} R1 = $\Sigma|F_o - F_c|/\Sigma F_o$ ^{b} wR2 = $\Sigma[w(F_o^2 - F_c^2)^2]/\Sigma[w(F_o)^2)]^{1/2}$

Table S2. Selective Interatomic Distances (Å) and Bonding Angles (°) of Au_4Ag_4 Cluster Complex **1**.

	interatomic distance		
Au1-Au1a	3.1980(7)		
Au1-Ag2	2.9464(7)	Au2-Ag1	2.9159(8)
Au1-Ag1	3.0269(7)	Au2-Ag2	2.8556(8)
Au1-C41	1.986(9)	Au2-C26	1.962(9)
Au1-C1	2.020(9)	Au2-C17	1.994(9)
Ag1-C40	2.701(8)	Ag2-C1	2.348(8)
Ag2-C2	2.625(9)	Ag2-C17	2.401(8)
Ag1-C26	2.357(10)	Ag1-C41	2.381(8)
	bonding angle		
Ag2-Au2-Ag1	129.26(2)	Ag2-Au1-Ag1	164.23(2)
Au2-Ag1-Au1	80.977(18)	Ag1-Au1-Au1	102.88(2)
Ag2-Au1-Au1	92.520(17)	Au2-Ag2-Au1	91.17(2)
C41-Au1-C1	174.3(4)	C26-Au2-C17	174.4(4)

Table S3. Selective Interatomic Distances (Å) and Bond Angles (°) of Au_4Ag_4 Cluster Complex **2**.

	interatomic distance		
Au1-Au1a	3.2384(7)		
Au1-Ag1	2.9963(6)	Au2-Ag1	2.8455(6)
Au1-Ag2	3.0488(6)	Au2-Ag2	2.8853(5)
Au1-C1	1.998(5)	Au2-C17	1.994(5)
Au1-C26	2.005(5)	Au2-C49	1.995(5)
Ag1-C26	2.372(4)	Ag2-C1	2.386(4)
Ag1-C49	2.428(4)	Ag2-C2	2.641(4)
Ag1-C27	2.618(4)	Ag2-C17	2.363(5)
	bond angle		
Ag1-Au2-Ag2	135.120(13)	Au2-Ag1-Au1	87.214(17)
Ag1-Au1-Ag2	162.989(12)	Ag1-Au1-Au1	95.283(17)
Ag2-Au1-Au1	101.564(17)	Au2-Ag2-Au1	79.815(17)
C1-Au1-C26	174.1(2)	C49-Au2-C17	174.18(19)

Table S4. The Partial Molecular Orbital Compositions (%) by SCPA Approach in the Ground State and the Absorption Transitions for Complex **1** in CH_2Cl_2 Solution, Calculated by TD-DFT Method at the PBE1PBE-GD3 Level.

orbital	energy (eV)	MO contribution (%)				
		Au (s/p/d)	Ag (s/p/d)	two L	other two L	PPh_3
LUMO+3	-1.82	11.35 (7/86/7)	11.53 (27/66/7)	41.11	34.67	1.34
LUMO+2	-1.83	31.96 (67/29/4)	25.76 (65/34/1)	27.42	13.49	1.36
LUMO+1	-1.87	8.58 (43/51/6)	25.83 (57/41/3)	28.87	34.08	2.63
LUMO	-1.96	13.43 (6/88/6)	24.81 (48/50/2)	15.87	39.53	6.35
HOMO	-5.53	8.21 (15/19/66)	11.02 (7/17/76)	65.75	6.82	8.20
HOMO-2	-5.76	23.34 (22/66/12)	8.82 (26/56/17)	63.23	1.34	3.26
HOMO-3	-5.85	16.80 (12/67/21)	6.35 (44/34/22)	1.68	73.79	1.37

state	*E*, nm (eV)	O.S.	transition (contrib.)	assignment	measured (nm)
S_1	420 (2.95)	0.0194	HOMO→LUMO (62%)<br>HOMO→LUMO+2 (23%)	^{1}LLCT/^{1}MC/^{1}IL<br>^{1}LMCT/^{1}IL/^{1}MC	443
S_5	405 (3.06)	0.4018	HOMO→LUMO+1 (59%)<br>HOMO→LUMO+3 (26%)	^{1}IL/^{1}LLCT/^{1}MC<br>^{1}IL/^{1}LLCT/^{1}MC	424
S_{10}	385 (3.22)	0.4338	HOMO-3→LUMO (29%)<br>HOMO-2→LUMO (29%)<br>HOMO-2→LUMO+2 (28%)	^{1}IL/^{1}MC/^{1}LMCT<br>^{1}LLCT/^{1}MC/^{1}IL<br>^{1}MC/^{1}IL/^{1}LMCT	

Table S5. The Partial Molecular Orbital Compositions (%) by SCPA Approach in the Lowest-Energy Triplet State and the Emission Transitions for Complex **1** in CH_2Cl_2 Solution, Calculated by TD-DFT Method at the PBE1PBE-GD3 Level.

orbital	energy (eV)	MO contribution (%)				
		Au (s/p/d)	Ag (s/p/d)	two L	other two L	PPh_3
LUMO+1	-1.90	15.47 (57/38/5)	27.46 (65/33/2)	15.19	38.98	2.88
LUMO	-2.05	19.59 (26/68/6)	25.72 (44/53/3)	37.13	12.18	5.38
HOMO	-5.34	7.34 (11/20/69)	11.05 (11/18/71)	73.55	1.70	6.36

state	*E*, nm (eV)	O.S.	transition (contrib.)	assignment	measured (nm)
T_1	659 (1.88)	0.0000	HOMO→LUMO (52%)<br>HOMO→LUMO+1 (11%)	^{3}IL/^{3}LMCT/^{3}MC<br>^{3}IL/^{3}LMCT/^{3}MC	650

Table S6. The Partial Molecular Orbital Compositions (%) by SCPA Approach in the Ground State and the Absorption Transitions for Complex **2** in CH_2Cl_2 Solution, Calculated by TD-DFT Method at the PBE1PBE-GD3 Level.

orbital	energy (eV)	MO Contribution (%)				
		Au (s/p/d)	Ag (s/p/d)	two L	other two L	PPh_2Py
LUMO+3	-1.82	26.26 (12/78/11)	18.25 (37/54/9)	23.13	19.73	12.63
LUMO+1	-1.89	26.53 (63/33/4)	28.56 (64/35/1)	36.35	6.48	2.09
LUMO	-1.99	23.05 (69/28/3)	26.22 (66/32/2)	5.70	40.09	4.94
HOMO	-5.58	9.53 (9/26/64)	8.41 (22/15/63)	61.40	15.23	5.42
HOMO-1	-5.70	14.08 (38/34/29)	12.14 (29/20/51)	14.36	52.15	7.28
HOMO-2	-5.82	25.58 (12/75/13)	9.97 (40/49/11)	32.80	29.49	2.16
HOMO-3	-5.84	20.63 (59/23/18)	6.00 (14/58/28)	34.94	36.28	2.16

state	*E*, nm (eV)	O.S.	transition (contrib.)	assignment	measured (nm)
S_1	417 (2.97)	0.0269	HOMO→LUMO+1 (47%)<br>HOMO→LUMO (37%)<br>HOMO-1→LUMO+1 (10%)	^{1}LMCT/^{1}IL/^{1}MC<br>^{1}LMCT/^{1}LLCT/^{1}MC<br>^{1}LMCT/^{1}MC/^{1}LLCT	445
S_5	395 (3.14)	0.3837	HOMO-2→LUMO (28%)<br>HOMO-3→LUMO (25%)<br>HOMO-1→LUMO+3 (17%)	^{1}MC/^{1}IL/^{1}LMCT<br>^{1}IL/^{1}MC/^{1}LMCT<br>^{1}IL/^{1}MC/^{1}LMCT	424
S_9	383 (3.24)	0.4324	HOMO-2→LUMO+1 (41%)<br>HOMO-3→LUMO+1 (29%)<br>HOMO-3→LUMO (15%)	^{1}MC/^{1}IL/^{1}LMCT<br>^{1}IL/^{1}LMCT/^{1}MC<br>^{1}IL/^{1}MC/^{1}LMCT	

Table S7. The Partial Molecular Orbital Compositions (%) by SCPA Approach in the Lowest-Energy Triplet State and the Emission Transitions for Complex **2** in CH_2Cl_2 Solution, Calculated by TD-DFT Method at the PBE1PBE-GD3 Level.

orbital	energy (eV)	MO contribution (%)				
		Au (s/p/d)	Ag (s/p/d)	two L	other two L	PPh_2Py
LUMO	-2.08	15.96 (20/72/8)	22.75 (46/51/2)	49.59	6.79	4.91
HOMO	-5.42	8.54 (6/28/66)	8.86 (17/23/60)	74.92	3.52	4.17

state	*E*, nm (eV)	O.S.	transition (contrib.)	assignment	measured (nm)
T_1	656 (1.89)	0.0000	HOMO→LUMO (58%)	^{3}IL/^{3}LMCT/^{3}MC	630

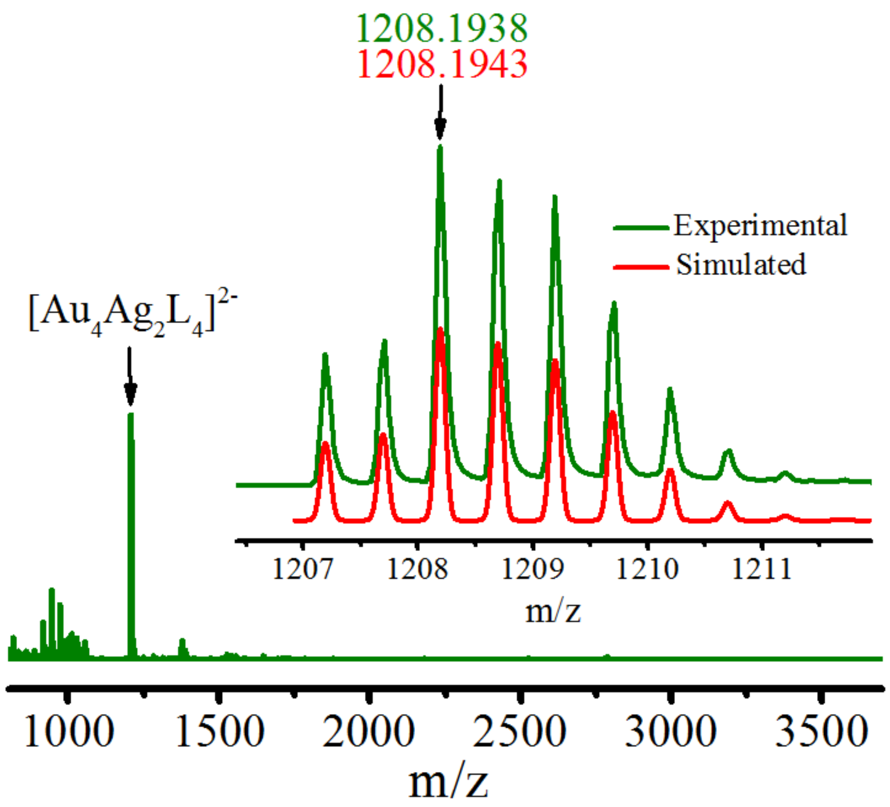

Figure S1. The high-resolution mass spectrometry of complex **1**. Inset: The measured and simulated isotopic patterns.

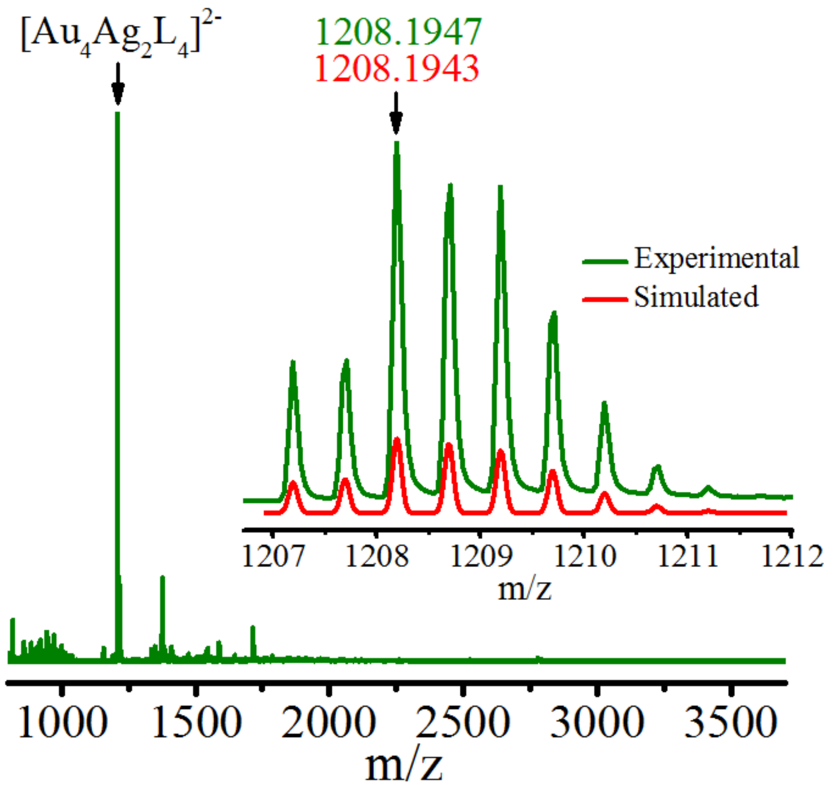

Figure S2. The high-resolution mass spectrometry of complex **2**. Inset: The measured and simulated isotopic patterns.

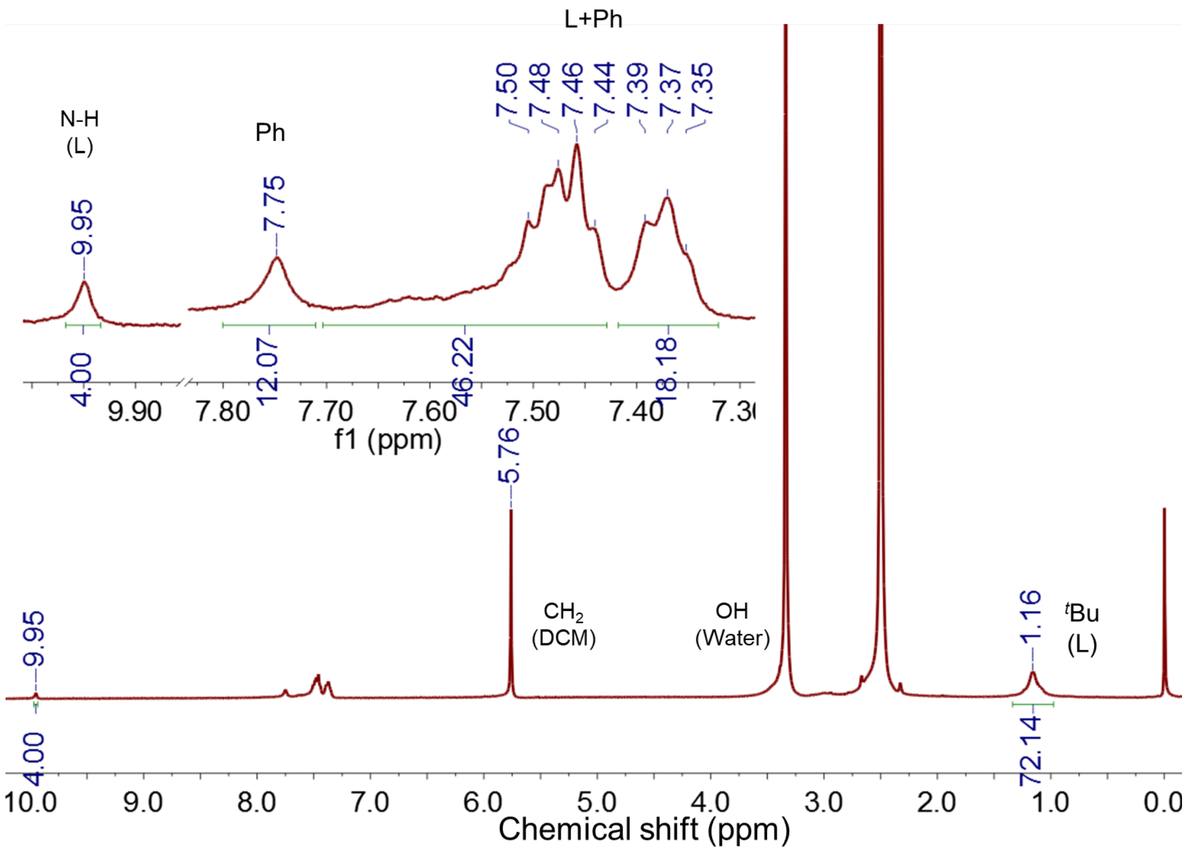

Figure S3. The ^{1}H NMR spectrum of complex **1** in DMSO-d_6 solution at ambient temperature.

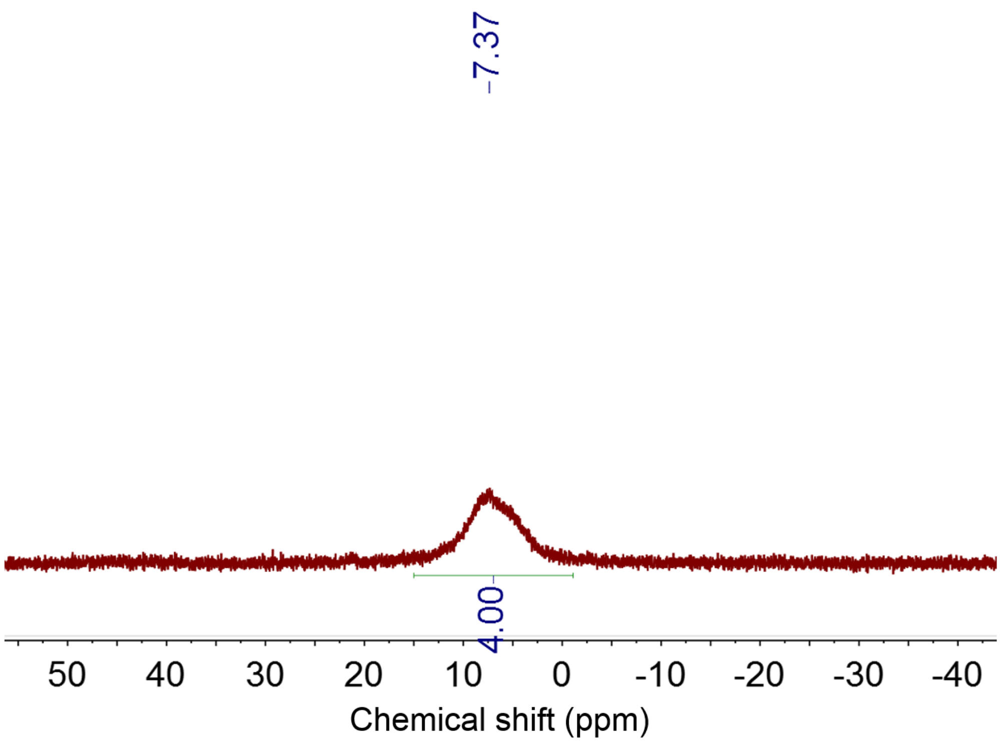

Figure S4. The ^{31}P NMR spectrum of complex **1** in DMSO-d_6 solution at ambient temperature.

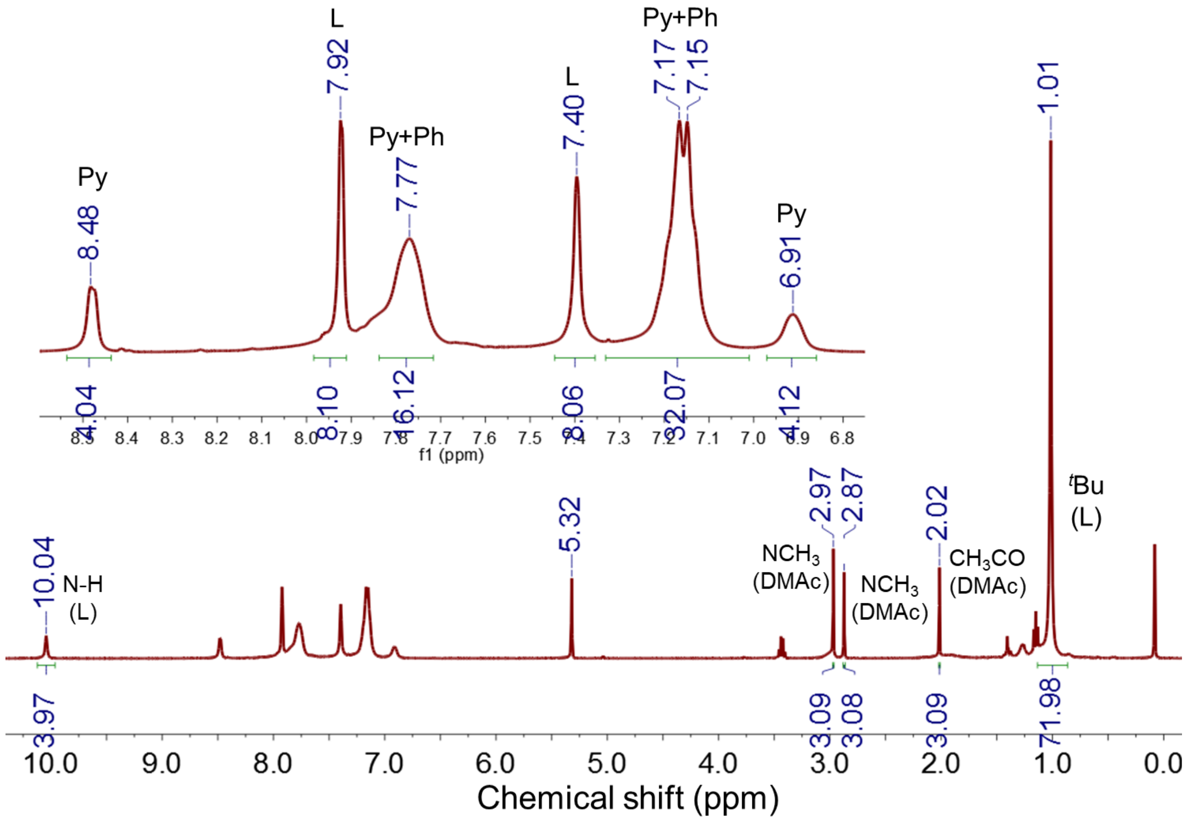

Figure S5. The ^{1}H NMR spectrum of complex **2** in CD_2Cl_2 solution at ambient temperature.

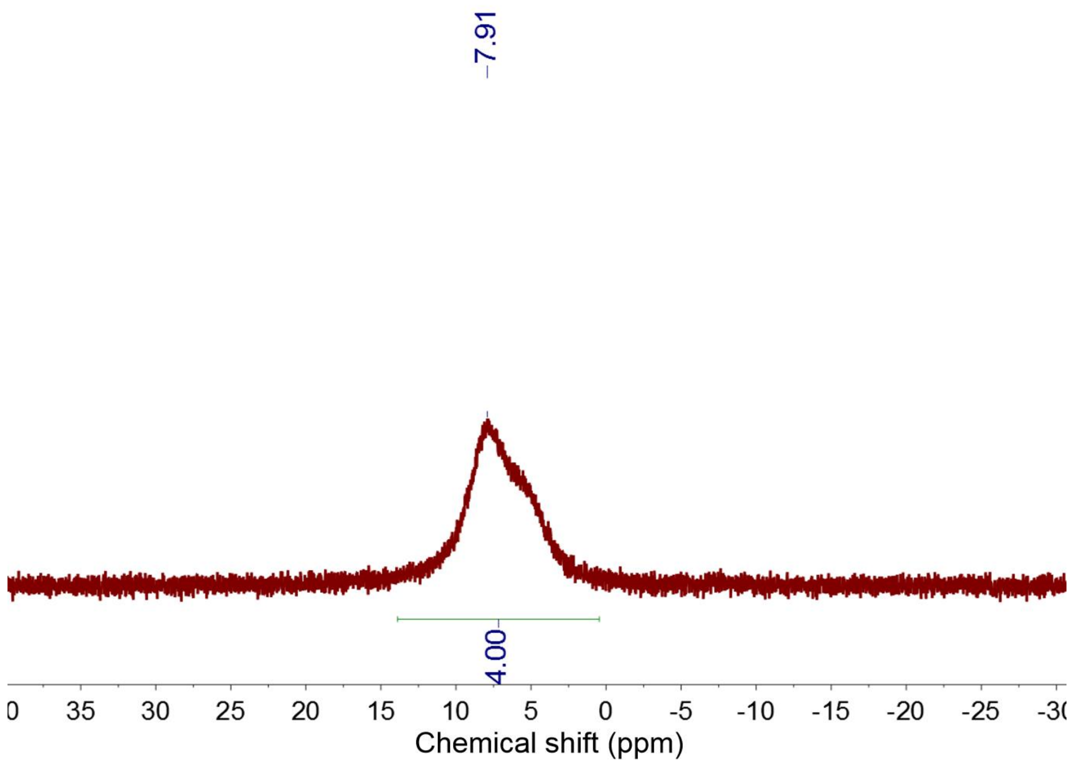

Figure S6. The ^{31}P NMR spectrum of complex **2** in CD_2Cl_2 solution at ambient temperature.

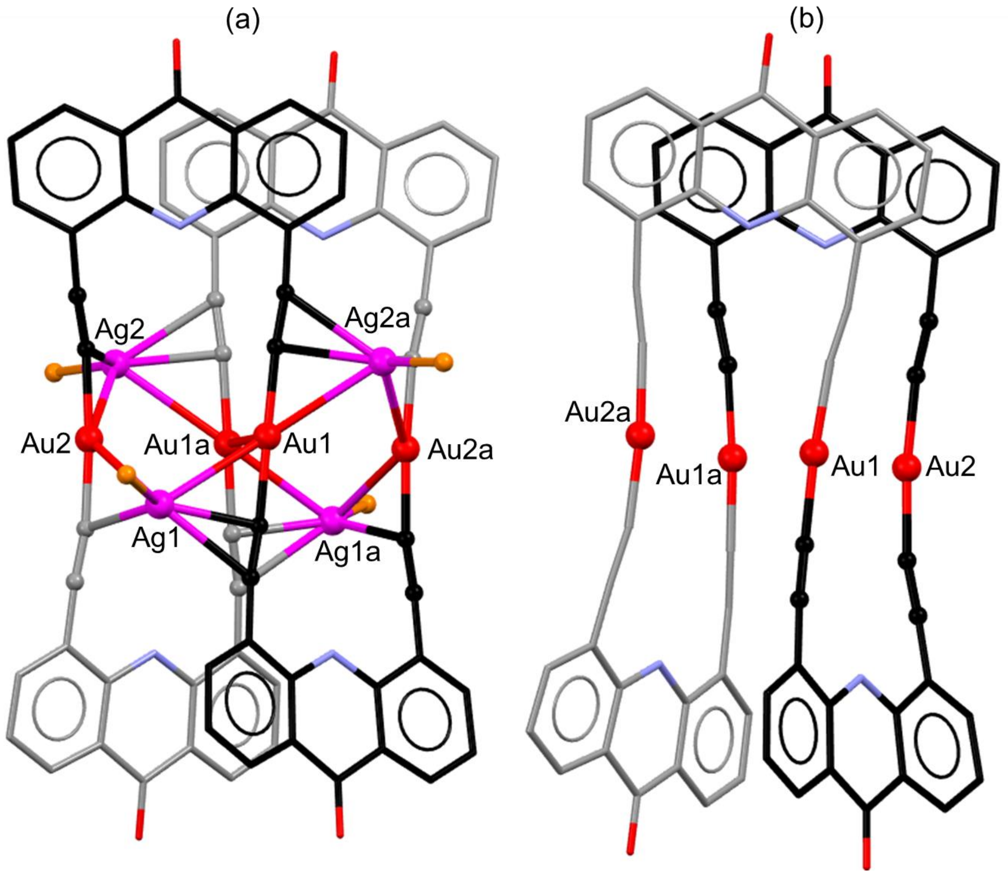

Figure S7. (**a**) A perspective view of Au_4Ag_4 complex **2** plotted from X-ray crystallography. The hydrogen atoms and *tert*-butyl groups together with the phenyl and 2-pyridyl rings on phosphorous atoms were omitted for clarity. (**b**) A view showing a twisted paper clip structure of gold(I)-bis(acetylide) coordination framework.

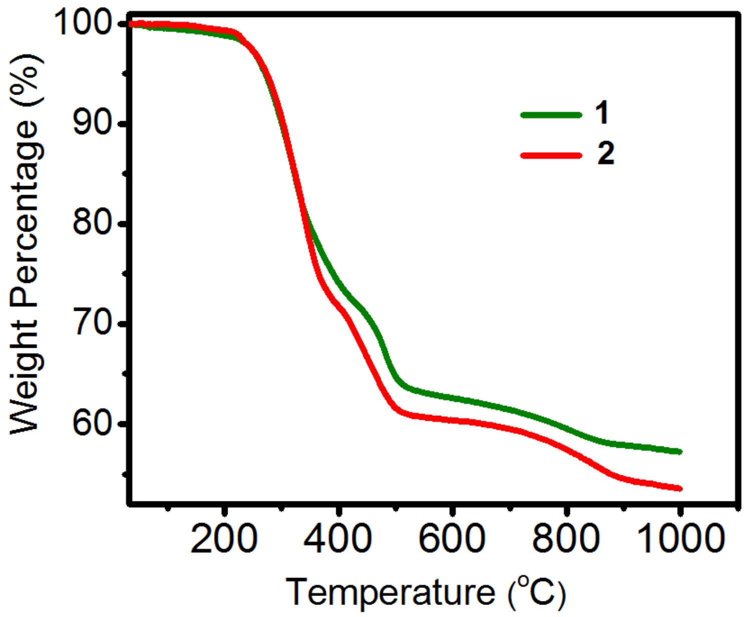

Figure S8. The plots of thermogravimetric analyses of complexes **1** and **2**.

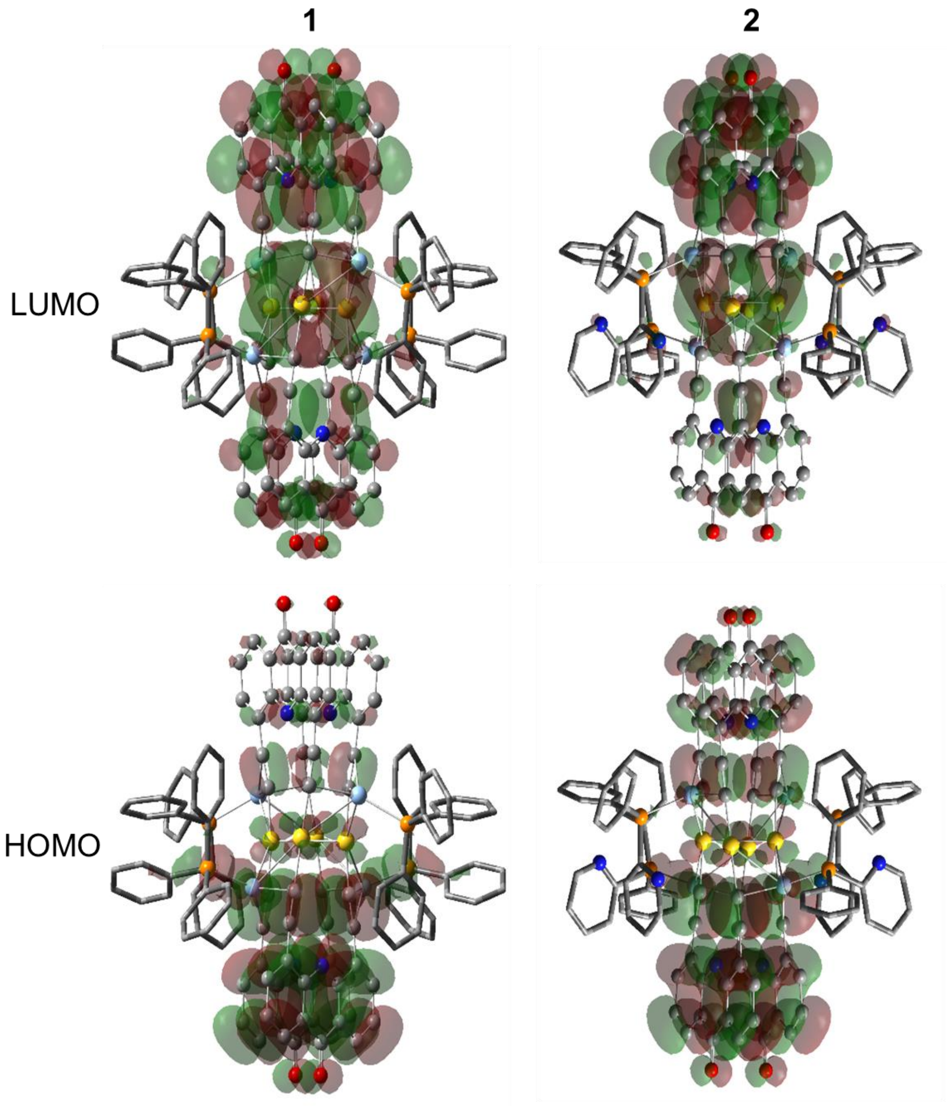

Figure S9. Plots of the HOMO and LUMO in the ground state for Au_4Ag_4 complexes **1** and **2** by TD-DFT method at the PBE1PBE-GD3 level.

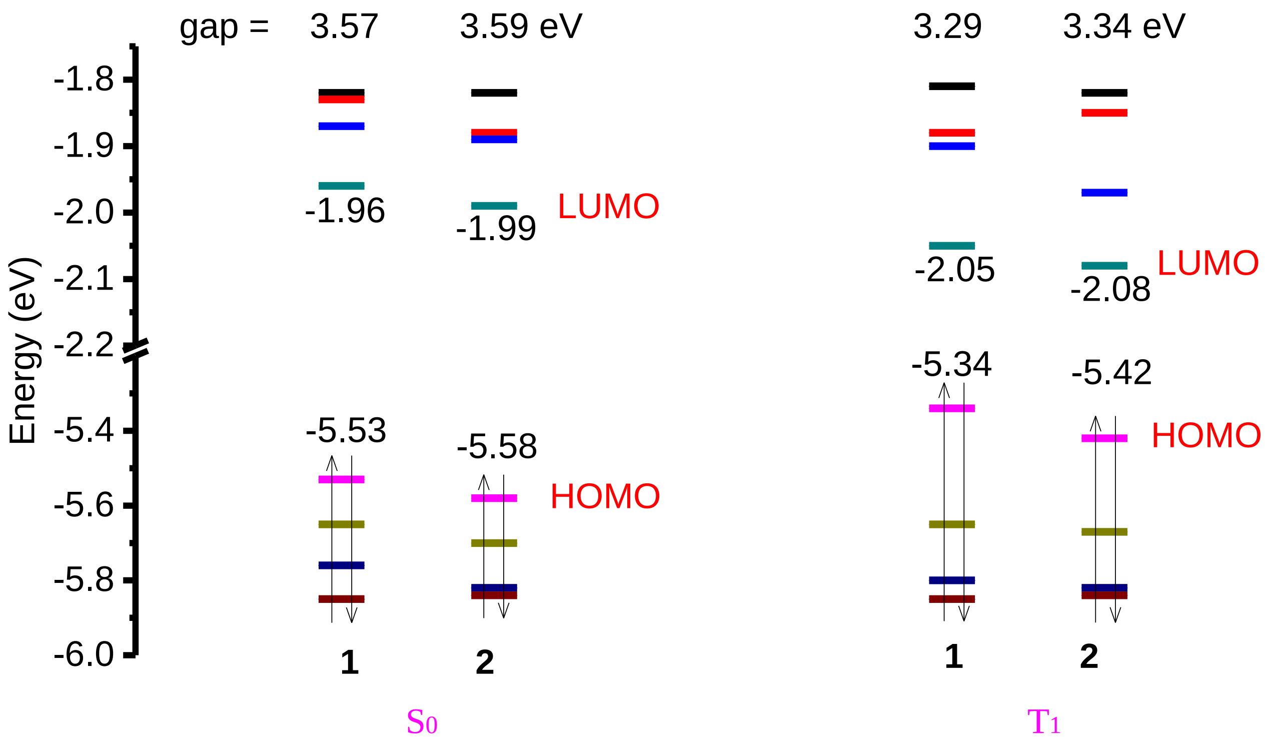

Figure S10. Plots of the energy level of frontier molecular orbitals (HOMO-3 ~ LUMO+3) based on the ground (S_0) and lowest-energy triplet (T_1) state structures of complexes **1** and **2**.

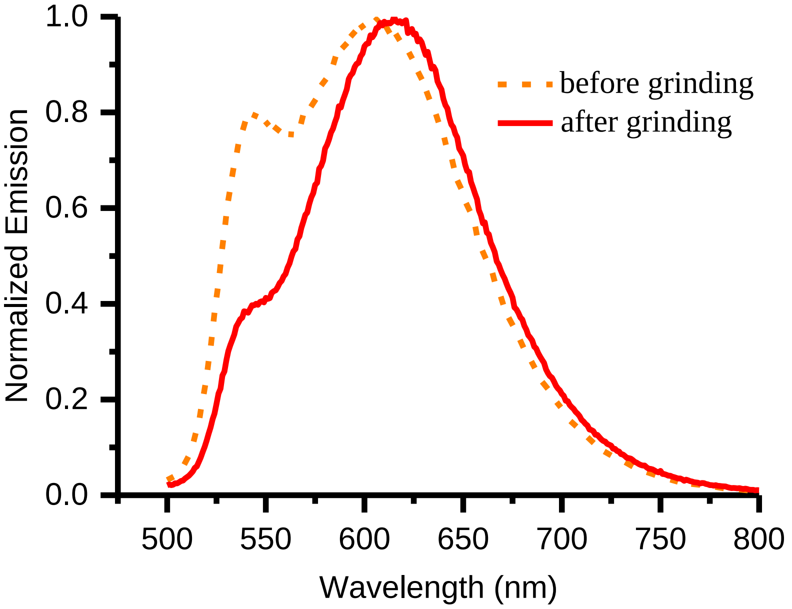

Figure S11. The normalized emission spectra of 3% Au_4Ag_4 cluster complex **1** in PMMA matrix under UV irradiation (365 nm) before and after mechanical grinding.

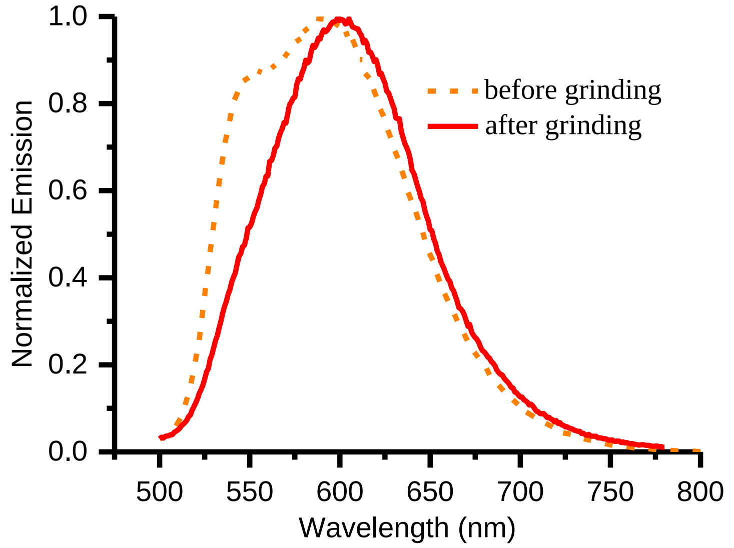

Figure S12. The normalized emission spectra of 3% Au_4Ag_4 cluster complex **2** in PMMA matrix under UV irradiation (365 nm) before and after mechanical grinding.

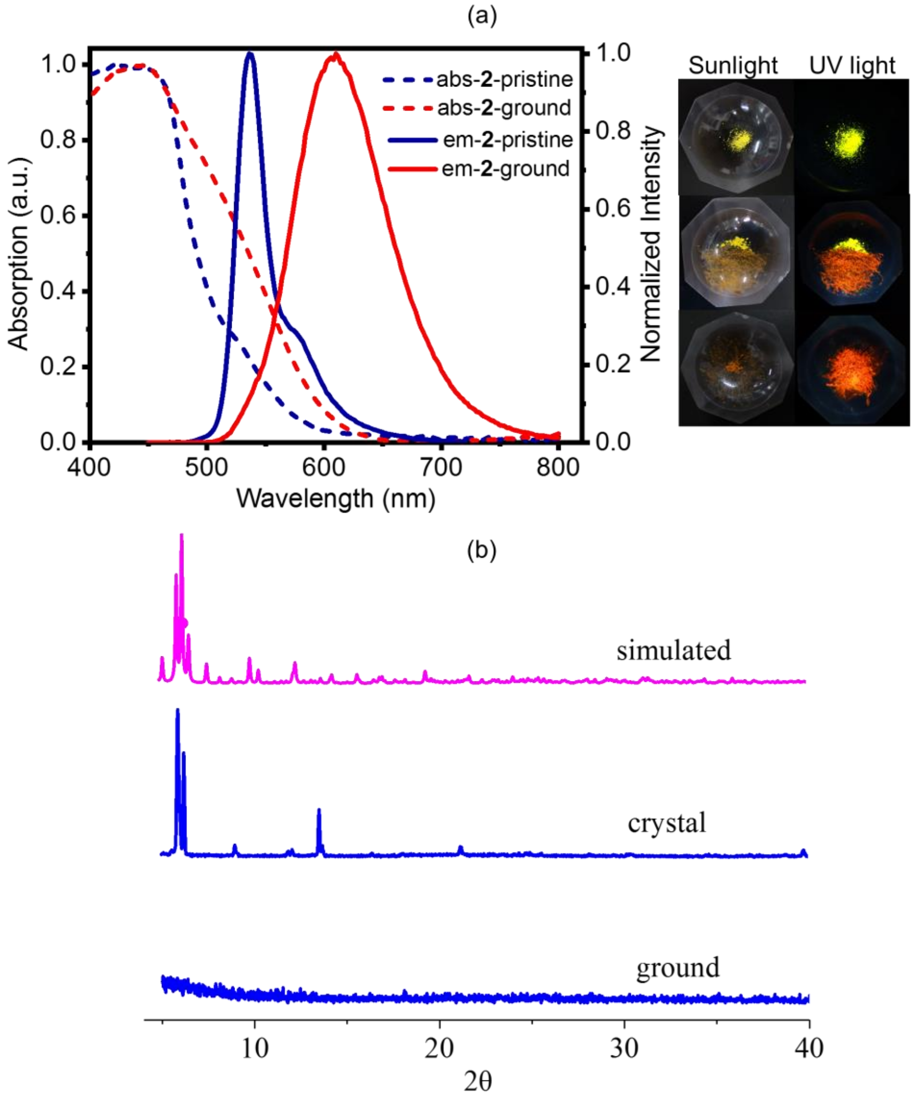

Figure S13. (**a**) The normalized UV-Vis absorption and emission spectra together with the images of Au_4Ag_4 cluster complex **2** under ambient light and UV irradiation (365 nm) before and after mechanical grinding. (**b**) The simulated and measured X-ray diffraction patterns of Au_4Ag_4 cluster complex **2** before and after mechanical grinding.
